# Improving monitoring of sexual, reproductive health, and rights globally

**DOI:** 10.1371/journal.pmed.1004525

**Published:** 2025-02-06

**Authors:** Sacha St-Onge Ahmad, Zulfiqar A. Bhutta

**Affiliations:** Centre for Global Child Health, Hospital for Sick Children, Toronto, Canada

The inclusion of sexual health and reproductive health targets in the Sustainable Development Goals (SDGs) aimed to provide impetus for tracking progress and advocating for sexual health and reproductive health rights for girls and women globally. With a rapidly changing political landscape in many countries, especially the United States, and with regions experiencing prolonged poly-crises, such as in the Middle East and North Africa region, it is imperative to ensure the tools used to monitor progress are valid and adaptable to varying contexts. SDG 5.6.2, defined as the “Number of countries with laws and regulations that guarantee full and equal access to women and men aged 15 years and older to sexual and reproductive health care, information, and education,” has potential to address this need.

In a new study, Jewel Gausman and colleagues [1] examine the validity of SDG 5.6.2’s current method of calculation by using country-level data to compare it to their revised version. This new version offers a potential resolution to 2 main concerns raised in the literature: one, that the indicator is sensitive to the number of barriers and enablers included, and two, that the overall score is a mean of the number of components rather than of the substantive domains [2]. The revised formula addresses these challenges by re-expressing barriers as the absence of enablers and by assigning the 4 substantive domains equal weightage.

Although this represents progress and an advancement, some issues remain. First, a country with multiple restrictions based on age, marital status, and third-party authorization for emergency contraception, for example, should be assigned a different score compared to a country with only limited restrictions. The revised formula continues to treat the impact of any one of these restrictions equal to the impact of all of them acting simultaneously on access to health care, information, and education. Second, a plural legal system (defined as a legal system in which multiple sources of law coexist) is listed as a barrier in at least 1 component of each section (e.g., Section 1, Component 1). While it is often the case that women fare worse under plural legal systems, it has been noted that they can and have conversely benefited from them by leveraging the operational ambiguity that accompanies the same systems [3]. Existence of a plural legal system on its own should be reconsidered as a barrier only when contradictory sexual health and reproductive health laws exist therein. Lastly, financing ought to be considered as a key potential barrier to the availability of the 13 commodities listed in Section 1, Component 2: Maternity Care, Life Saving Commodities.

Though a common measure is useful for cross-country comparisons, SDG 5.6.2 is particularly susceptible to between- and within-country variations. Additional guidance to countries on adapting the proposed new measure to local contexts and in certain humanitarian settings could increase its acceptability and application within countries. Below, we share 2 examples of when adaptation may present an improvement and enhance the utility of the measure within countries.

First, the option to customize the measure to make it more locally relevant under any circumstance could resolve the following 2 potential problems: (1) the proposed list of types of barriers and enablers in each component are broad but still may not cover all contexts; and (2) the underlying assumption of the measure that all countries are striving to achieve full and equal access to sexual health and reproductive health rights sets a global standard without accounting for variations in cultural values (most relevant for Section 3: Sexuality Education).

Second, growing evidence on the impact of conflict, displacement, and climate change on girls’ and women’s sexual health and reproductive health outcomes [4,5] suggests a strong and time-sensitive need to develop appropriate measures to monitor SDG 5.6.2 in varied settings, especially fragile humanitarian contexts. Promoting self-care interventions in sexual health and reproductive health as a strategy to both empower girls and women and expeditious care seeking and provision could help overcome challenges in fragile health systems exacerbated during crises. The recent guidelines on self-care interventions for sexual and reproductive health and rights published by the World Health Organization [6] offer a starting point for integrating these within health systems and developing monitoring tools, a process which some countries have already begun [7]. The proposed measure could be improved for use in humanitarian contexts if it incorporated a component that captured self-care strategies. Further, building on existing mechanisms to track data for the measure, a database like Women, Business, and the Law [8] that tracks the status of each component each year by country would further enhance its potential impact.

Lastly, with political landscapes increasingly shifting towards national conservatism in many countries [9] that have historically made among the largest donor contributions to global family planning initiatives [10], implementation strategies and financing of programs that progress SDG 5.6.2 is a key consideration. A companion indicator to monitor domestic investments and donor assistance for family planning is an important parameter that should be incorporated into the mix of indicators used to monitor sexual health and reproductive health progress worldwide.
